# UNderstanding uptake of Immunisations in TravellIng aNd Gypsy communities (UNITING): protocol for an exploratory, qualitative study

**DOI:** 10.1136/bmjopen-2015-008564

**Published:** 2015-06-08

**Authors:** Cath Jackson, Helen Bedford, Louise Condon, Annie Crocker, Carol Emslie, Lisa Dyson, Bridget Gallagher, Susan Kerr, Helen J Lewis, Julie Mytton, Sarah A Redsell, Frieda Schicker, Christine Shepherd, Lesley Smith, Linda Vousden, Francine M Cheater

**Affiliations:** 1 Department of Health Sciences, University of York, York, UK; 2 Institute of Child Heath, UCL, London, UK; 3 College of Human and Health Sciences, Swansea University, Swansea, UK; 4 Formerly in the Gypsy and Traveller Team, Bristol City Council, Bristol, UK; 5 Institute for Applied Health Research, Glasgow Caledonian University, Glasgow, UK; 6 Formerly at South Glasgow Community Health Partnership, NHS Greater Glasgow & Clyde, Glasgow, UK; 7 Centre for Child and Adolescent Health, University of the West of England, Bristol, UK; 8 Faculty of Health, Social Care and Education, Anglia Ruskin University, Cambridge, UK; 9 Formerly at the London Gypsy and Traveller Unit, London, UK; 10 York Travellers Trust, York, UK; 11 Women and Children's Directorate, North Bristol NHS Trust, Bristol, UK; 12 School of Health Sciences, University of East Anglia, Norwich, UK

**Keywords:** gypsy, traveller, Roma, immunisation, vaccination

## Abstract

**Introduction:**

Gypsies, Travellers and Roma (referred to here as Travellers) experience significantly poorer health and have shorter life expectancy than the general population. They are also less likely to access health services including immunisation. To improve immunisation rates, we need to understand what helps and hinders individuals in these communities in taking up immunisations. This study has two aims: (1) Investigate the barriers and facilitators to acceptability and uptake of immunisations among six Traveller communities in the UK; (2) Identify potential interventions to increase uptake in these Traveller communities.

**Methods and analysis:**

A three-phase qualitative study with six Traveller communities. PHASE 1: In each community, we will explore up to 45 Travellers’ views about the influences on their immunisation behaviours and ideas for improving uptake in their community. PHASE 2: In each community, we will investigate 6–8 service providers’ perspectives on barriers and facilitators to childhood and adult immunisations for Traveller communities with whom they work, and ideas to improve uptake. Interview data will be analysed using the Framework approach. PHASE 3: The findings will be discussed and interventions prioritised in six workshops, each with 10–12 phase 1 and 3–4 phase 2 participants.

**Ethics and dissemination:**

This research received approval from NRES Committee Yorkshire and The Humber-Leeds East (Ref. 13/YH/02). It will produce (1) findings on the barriers and facilitators to uptake of immunisations in six Traveller communities; (2) a prioritised list of potentially feasible and acceptable interventions for increasing uptake in these communities; and (3) methodological development in undertaking research with diverse Traveller communities. The study has the potential to inform new ways of delivering services to ensure high immunisation uptake. Findings will be disseminated to participants, relevant UK organisations with responsibility for the implementation of immunisation policy and Traveller health/welfare; and submitted for publication in academic journals.

**Trial registration number:**

ISRCTN20019630.

**Strengths and limitations of this study**

- This is the first large-scale multi-community qualitative study exploring barriers and facilitators to childhood and adult immunisation for Travellers in the UK.
- The findings will have the potential to inform new ways of delivering accessible immunisation services to socially excluded, marginalised communities.
- Our research team may be perceived as ‘outsiders’, resulting in difficulties in engaging with Travellers to participate.

## Introduction

Travellers typically experience significantly poorer health and shorter life expectancy compared to the general population.^1–6^ (*Note*. Throughout this paper, we use the term Traveller in its broadest sense to include distinct and diverse Gypsy, Traveller and Roma communities, who may be settled or nomadic, and may live on authorised or unauthorised sites or in houses). Despite a greater health need, there is low uptake of health services by Travellers, including preventive healthcare.^1–6^ Barriers to uptake stem partly from a lack of consideration of Traveller culture by health providers when developing services, for example, a reluctance by general practitioner's (GP) practices to register transient Travellers.^7^ Further barriers are a history of discrimination leading to mistrust of people and institutions, poverty, low health literacy and, in some communities, strong beliefs of ‘stoicism’, ‘self-reliance’ and ‘fatalism’ about health.^7^

The public health benefits and cost-effectiveness of immunisation are well established.^8^ In the UK, the uptake rates for scheduled immunisations in children up to 6 years are generally high and stable, approaching or meeting the 95% target required for herd immunity.^9^ ^10^ Until the 2011 census,^11^ ethnic group classifications did not provide for Travellers to ‘self-identify’. Moreover, ethnic group is not routinely collected during registration with aGP, and even now may be incomplete or poorly coded.^12^ There is, therefore, a lack of accurate information about the take-up of immunisations in Traveller communities. That said, local studies using parent self-report^13–16^ and National Health Service (NHS) records^14^ ^17^ suggest low or variable uptake of childhood immunisation. These patterns mirror data for other disadvantaged groups which are more likely to be unimmunised or not up to date, significantly increasing their risk (and consequent spread) of vaccine preventable disease.^18^ ^19^

A large body of literature^20–24^ identifies two broad categories of parental factors influencing uptake of childhood immunisation in the general population and high-risk groups.^25^ The first relates to socioeconomic disadvantage where, despite being motivated to have their children vaccinated, parents lack access to resources and support to overcome logistical barriers such as having no private transport. The second relates to parents’ concerns about the safety or beliefs about the necessity of vaccines. There are differences in parents who accept immunisation but do not complete the course (partial immunisers), those who have concerns about the safety of some vaccines but not others (selective immunisers) and those who reject immunisation altogether (non-immunisers).^26^ These different groups are likely to require different interventions. Regardless of parental position on immunisation, trust in health professionals and services is paramount. Studies have also explored factors influencing uptake of immunisation in adults^27^ ^28^ including those with ‘high risk’ conditions^29^ and minority ethnic groups.^30^ The barriers appear to fall into the same two categories, access and beliefs, including the perception that healthy people do not need immunisations.^27^

Many of the issues identified in this literature are likely to be similar for Travellers. However to develop interventions that are tailored to the needs of diverse Traveller communities, further research with these communities is required. To date, a small number of studies^13–17^ have explored the barriers to immunisation uptake specifically in Traveller communities. These identify multiple issues reflecting the difficulties experienced by marginalised, socially excluded communities.^1–6^ However, these studies tend to be small and focused on one community. While Traveller communities may share similar features of lifestyle that distinguish them from the general population, they have different beliefs and cultural traditions.^31^ It is therefore important to understand whether and how specific communities differ in the factors that promote or inhibit immunisation. Second, immunisation is often only one small part of a study exploring several health issues with Travellers. This limits the extent to which the complex nature of barriers and facilitators to immunisation is explored. For example, barriers may be specific to particular vaccines, for example, measles, mumps rubella (MMR) vaccine, or differ for adult and childhood vaccines. Third, most studies were conducted in the 1980s/1990s, so do not consider issues associated with the introduction of new vaccines in the UK childhood immunisation schedule (eg, Rotavirus in July 2013^32^), evolving views about previously controversial vaccines (eg, pertussis, MMR) or the views of more recent migrant communities in the UK, for example, Romanian Roma. Finally, we have found no studies on immunisation uptake in adults living in Traveller communities.

The effectiveness of interventions to increase immunisation uptake among children^33^ and adults^34^ ^35^ has also been reviewed and there are many examples of innovative health and social care provision aimed at improving the health of Travellers.^1^ ^2^ ^36^ Some target immunisation specifically (eg, outreach immunisation programmes, tailored health promotion resources), whereas others are generic yet relevant to immunisation (hand-held patient records, specialist health visitors^1^ and cultural competence training of health professionals^37^). These interventions are rarely rigorously evaluated, so it is unclear which are feasible, acceptable and (cost) effective, in which communities they work and how they may (not) work. Finally, existing interventions are rarely informed by theoretical frameworks which can increase effectiveness by aiding understanding of the likely mechanisms of change.^38^ Our research will advance understanding by addressing the limitations of previous research. The aims and objectives are as follows.

### Aims

1. Investigate the barriers and facilitators to acceptability and uptake of immunisations among six Traveller communities (comprising five distinct ethnic/cultural groups) across four UK cities;
2. Identify possible interventions to increase uptake of immunisations in these Traveller communities, which could be tested in a subsequent feasibility study.

### Objectives

1. Investigate the views of Travellers on the barriers and facilitators to acceptability and uptake of immunisations and explore their ideas for improving immunisation uptake;
2. Examine whether and how these responses vary across and within communities, and for different vaccines (childhood and adult);
3. Investigate the views of service providers on the barriers and facilitators to uptake of immunisations within the Traveller communities with whom they work, and explore their ideas for improving immunisation uptake;
4. Examine whether and how these responses vary within and across communities, for different vaccines (childhood and adult) and for different service provider roles;
5. Use the data collected from (1 to 4) to identify possible interventions to increase uptake of immunisations in the six Traveller communities;
6. Conduct Feedback Workshops in each community to discuss findings and to produce a prioritised list of potentially feasible and acceptable interventions to be considered for testing in a subsequent feasibility study.

## Methods and analysis

### Study design

This three-phase qualitative study is the first ‘intervention development’ stage of the Medical Research Council Framework for developing and evaluating complex interventions.^38^ Phase 1 comprises qualitative semistructured, group and individual interviews in six Traveller communities. Phase 2 comprises qualitative semistructured individual interviews with service providers. In phase 3, Feedback Workshops will be held with each Traveller community and associated service providers to produce a prioritised list of potentially feasible and acceptable interventions for future development and testing. The theoretical framework underpinning the study is the Social Ecological Model (SEM, ref. 39) which recognises that individuals’ behaviour is affected by, and conversely impacts on, multiple levels of influence (intrapersonal, interpersonal, institutional, community and policy). The SEM has previously been used in the context of flu immunisation,^40^ child health^41^ and with culturally diverse^42^ and disadvantaged populations.^43^

### Setting and participants

#### Setting

The study will focus on six Traveller communities and be undertaken in four UK cities (York, Bristol, Glasgow and London (see table 1). Five of the communities (English Roma, English Gypsies, European Roma, Irish Traveller) were originally recognised in the Race Relations Act 1976 as ethnic minorities,^44^ replaced now by the 2010 Equality Act.^45^ While they have different beliefs, customs and languages, they share common features of lifestyle and culture^44^ and are genealogically and linguistically related.^46^ In contrast, Showpeople are not recognised in these Acts or perceived by the aforementioned communities as being part of the ‘traditional Travellers’ ethnic group. We include Eastern European Roma communities in Bristol and Glasgow because this is the newest and most under-researched Traveller community in the UK. In addition, these are communities that have travelled from different countries (Slovakia or Romania), speak different languages (Slovakian Roma or Romanian Roma) and therefore cannot be assumed to be similar.

**Table 1:** Overview of participating Traveller communities and examples of service providers linked to these communities (at the start of the study)

City	Community	Overview^46^
York	English Roma	Recognised in British Law as an ethnic group. 350+ families living across three official sites (54 pitches) and some in housing. *Examples of organisations/workers:* City of York Council-Lead for Traveller and Ethnic Minority Services, Traveller Support Worker; Joseph Rowntree Foundation, NHS York (moving to City of York Council in 2013)—Lead for Traveller Health, Health professionals based at GP practices close to the three official sites, Health visitor who worked at Personal Medical Service for Homeless People and Travellers Families project in York. This Service closed in 2011
Bristol	Eastern European Roma	Descended from the same people as British Romany Gypsies and have recently moved to the UK from Central and Eastern Europe. Recognised as the same ethnic category as British Gypsies yet distinct from the UK community. 40 families in shared rented accommodation in relative proximity to each other. *Examples of organisations/workers:* Bristol, North Somerset and South Gloucestershire Strategic Group for Traveller Health, Immunisation leaders in Bristol NHS, local Health Protection Unit, Bristol City Council Gypsy and Traveller team, designated Health Visitor, Roma worker funded by the church where the drop-in is located
	English Gypsy	Recognised in British Law as an ethnic group. 100+ families living on two council managed Traveller sites. *Examples of organisations/workers:* As for Eastern European Roma
Glasgow	Eastern European Roma	See Eastern European Roma in Bristol for overview. Based on GP records, there are 1800 residents housed in a very small geographical area in Govanhill (8 streets). *Examples of organisations/workers:* One full-time health visitor and two support workers (one bilingual) who are employed to work solely with the Roma Community in Govanhill, health professionals at Govanhill Health Centre, Oxfam, Govanhill Housing Association, Daisy Street Neighbourhood Centre, Govanhill Law Centre, Glasgow Community Health Partnership
	Scottish Show People	Scottish showman or travelling show, circus and fairground families. Not recognised in British Law as an ethnic group. Approximately 300 live in fixed sites in the North East of Glasgow. Some sites are owned by the council and some are privately owned. *Examples of organisations/workers:* Glasgow Community Health Partnership, health professionals at local Health Centre
London	Irish Traveller	Traditionally nomadic people of Celtic descent who arrived in Britain in the 1850s. Recognised in British Law as an ethnic group.17 000 live in London. Most live in rented accommodation and on local authority sites. The London Gypsy and Traveller Unit works with approximately 800 families. *Examples of organisations/workers:* London Gypsy Traveller Forum, Greater London Authority Public Health Team, Irish Traveller Movement, Southwark Travellers Action Group

These organisations were identified prior to the NHS reforms in April 2013.

GP, general practitioner; NHS, National Health Service.

### Participants

*Phase 1:* In each Traveller community, we will recruit men and women living in extended families across generations. We will include young women planning families, parents and grandparents to capture a lifespan/cross-generational perspective. We will actively seek participants eligible for specific vaccines including teenage girls for views on their 3-in-1 teenage booster (diphtheria, tetanus, poliomyelitis; given at around 14 years of age) and human papilloma virus (HPV) vaccine (given at 12–13 years); and adults who are pregnant, over 65 years or have long-term conditions for views on flu and pertussis vaccines). Typically, decisions on childhood immunisation are made by mothers;^20^ ^21^ however, we are keen to recruit men and women to explore any potential gender differences in views; we will therefore aim for a quarter of participants to be male. We will purposively seek to recruit a mix of full immunisers/partial/selective immunisers and non-immunisers (based on self-report^26^). We aim to interview approximately 24 to 45 participants in each of the six Traveller communities (total 144–270 participants). This large sample size will enable us to look for potential differences and similarities in views about both childhood and adult vaccines *within* a community across gender and age as well as draw out meaningful comparisons *across* Traveller communities, to allow robust conclusions to be made.

*Phase 2:* Service providers in the four cities will be recruited to the study. We will purposively sample to ensure that we interview a mix of ‘frontline workers’ (eg, health visitors, practice nurses, community midwives, school nurses, GPs, range of community workers including the third sector) and those working in more strategic/commissioning roles (eg, local decision-makers in health protection/public health/Health and Wellbeing Boards/Clinical Commissioning Groups). We will aim to interview six to eight service providers in each city (total 24 to 32 participants). Examples of organisations and providers that we intend to approach are presented in table 1. These organisations were identified prior to the NHS reforms in April 2013.

*Phase 3:* A subsample of participants from phases 1 and 2 will be invited to take part in the ‘Feedback Workshops’, specifically between 10 and 12 Traveller participants per community and three to four service providers (13 to 16 participants in total per workshop; six workshops comprising 78 to 96 participants). Ideally, we will attract a mix of Traveller men and women, across ages (including teenage girls) with different experiences of immunisations; and a mix of frontline service providers and those with a more strategic role.

### Access and recruitment

This is a complex, multisite project working with socially excluded, marginalised communities who are traditionally hard to engage in research.^47^

*Phase 1:* In each community, we will use a multipronged approach to access and recruitment. We are aware that our position of researchers as ‘outsiders’ with few similarities of experience and no ‘network connections’ with participant Traveller communities could result in difficulties in gaining access.^47^ Our research team includes ‘comprehensive gatekeepers’^47^ who have long-standing relationships with the communities and who can ‘vouch’ for our trustworthiness and thereby enable access and help recruit participants to the study. The proposed approach is based on the experience of these gatekeepers as well as drawing on established good practice.^48^ We will attend existing groups where members of the community routinely meet together. We will also promote the study via the local ‘frontline workers’ and, where appropriate, accompany them on visits to Traveller sites. We will meet with five or six members of each community throughout the study to inform the research process including recruitment. These ‘Community Partners’ will help ensure that issues such as access, recruitment and dissemination methods are acceptable. ‘Community Partners’ will be offered a £40 gift voucher of their choice per meeting in line with recommended payment for University public involvement groups.^49^

The study will be discussed with members of the community and written information provided for people to read or have read to them by others to aid discussions with family members and peers. For the two European Roma communities, information will be in English and Romanian/Slovakian. We will return a week later to establish who might wish to take part, and to arrange a time and place for the interview. We will regularly meet with the community during the recruitment phase.

*Phase 2:* In each city, we will establish a list of contacts of the relevant service providers. We will approach these people by email, in the first instance, with a participant information sheet (PIS) and then follow this up with a telephone call a week later. Our existing links to the multiple agencies working in each city will also be used to facilitate introductions.

*Phase 3:* Participants in phases 1 and 2 who agree to be contacted about phase 3 will be reapproached using their preferred method of contact and provided with a PIS about the Feedback Workshop. Where insufficient phase 1 participants are able to attend, we will seek to recruit new members of the community to attend the workshop.

### Data collection

*Phase 1:* We will conduct small group interviews with members of the same family to elicit a cross-generational/lifespan perspective. We also plan to run group interviews with teenage girls, and with young women planning families, pregnant women and those with preschool children, to capture peer influence on immunisation decisions. While immunisation may not seem a particularly sensitive issue (with the exception of HPV), group interviews may not be appropriate or favoured. We will, therefore be flexible about whom and how many participants take part in an interview. Individual and group interviews will be conducted face to face in participants’ choice of setting. With the consent of participants, interviews will be recorded digitally and transcribed verbatim. Interpreters will be employed for Roma interviews.

*Phase 2:* We will conduct individual interviews with the service providers face to face in participants’ choice of setting. With their consent, interviews will be recorded digitally and transcribed verbatim.

In phases 1 and 2, we will use topic guides for the interviews to ensure consistency; however, the format will be flexible to allow participants to generate naturalistic data on topics they view as important. We will explicitly pursue negative cases (‘elements in the data that appear to contradict the emerging view’) (ref. 50, p. 51) to enhance the validity of our developing propositions. Topics will be revised as necessary on the basis of emerging evidence from preceding interviews. The SEM^39^ will inform the questions that we ask, ensuring that we explore all five levels of influence on immunisation behaviour. Research team members’ local knowledge of immunisation and the Traveller community will also feed into the development of topic guides to prompt discussion of particular local issues (eg, outbreaks of measles, introduction/removal of specialist services). Topic guides will be reviewed and piloted with the ‘Community Partners’.

At the end of phases 1 and 2, for each of the six Traveller communities, we will (1) understand the potential barriers and facilitators for take-up of immunisations (across all five levels of the SEM) and (2) have ideas for the content and delivery of potentially feasible and acceptable interventions to increase immunisation uptake for all five levels of the SEM. We will have insight into whether the barriers and facilitators and interventions are similar or different dependent on the gender, age and self-reported immunisation history of Traveller participants, the professional role of service providers and across different vaccines (childhood/adult). These outputs will be presented at the Feedback Workshops in Phase 3.

*Phase 3:* A Feedback Workshop will be held locally for each Traveller community. The aim of the workshops is to disseminate the findings of phases 1 and 2 and to discuss and ‘co-produce’^47^ ideas for the content and delivery of potentially feasible and acceptable interventions at all five levels of the SEM. Following the presentation of the findings, we will use a structured two-step process.^51^ First, participants will independently rate suggested interventions for potential impact. Using these ratings, Travellers and service providers will then work together to jointly agree on a prioritised list of potentially feasible and acceptable interventions which could positively impact on immunisation uptake in their community.

### Data analysis

#### Within-community

*Phases 1 and 2*: Interviews will be fully transcribed and data subjected to thematic analysis using the Framework approach,^52^ which is designed to address applied policy-related questions.^52^ The Framework approach has previously been used in a large UK qualitative study exploring health issues with Gypsies and Travellers.^53^

The five stages of analysis, specified below, will first be undertaken independently for each of the six Traveller communities and for both phase 1 and phase 2 data. This ‘within-community’ analysis will be led by CJ, LD and CE. Members of the research team based in each city will contribute at stages 2 and 5. This will enhance rigour and ensure that the local context in which the data are collected and where participants live is appropriately considered. NVivo software will facilitate data management.

1. *Familiarisation*—The interview transcripts will be read and emerging ideas and recurrent themes recorded.
2. *Identifying a thematic framework*—A thematic framework will be set up in which the interview data will be organised. This will be informed by the SEM. Additional themes representing emerging issues from the data will be added. The framework will be applied to three to four transcripts to refine. The same thematic framework will be used to analyse the data for all the six Traveller communities and associated service providers.
3. *Indexing*—The thematic framework will be systematically applied to the interview data.
4. *Charting—*Charts will be drawn up for each theme and summaries of responses from participants (and verbatim quotes) entered. This will enable us to consider the range of views within each theme, that is, on (1) barriers, (2) facilitators to immunisation, (3) ideas for intervention across the five levels of the SEM; and (4) other emerging issues.
5. *Mapping and interpretation—*The charts will be reviewed and interrogated to compare and contrast views, as well as to seek patterns, connections and explanations within the data.

### Cross-community synthesis

The final outputs of the analysis of phase 1 and 2 data will be a thematic synthesis that takes account of the inferences derived from all the interview data for the sample as a whole.^54^ Using the charts created in stage 4 of the analysis for each community (both Travellers and service providers), we will synthesise the data across all six communities to explore similarities and differences in views on the same four areas: (1) barriers; (2) facilitators to immunisation; (3) ideas for intervention across the five levels of the SEM; and (4) other emerging issues. At this stage, we will also look for similarities and differences in views of the two European Roma communities living in different cities. Our earlier analysis exploring ‘within-community’ (eg, gender) patterns of responses will be extended across communities to enable us to identify the transferability of these features across communities.

## Ethics and dissemination

### Ethics

This research has received approval from NRES Committee Yorkshire and The Humber—Leeds East (Ref. 13/YH/02). Drawing on the British Psychological Society's Code of Human Research Ethics,^55^ there are three key ethical issues. First (principle of social responsibility^55^), it is a qualitative study with the primary output being a list of prioritised interventions to increase uptake for testing in a subsequent research study, rather than making actual changes to services. There is, therefore, a risk that we will raise unrealistic expectations of what this research can achieve in the short term with a lack of tangible benefits to the Traveller communities themselves, particularly those members who are transient. We will be very clear from the outset about the purpose of the research and work with our ‘Community Partners’ throughout the study to ensure that this stated purpose is widely disseminated. All Traveller participants will be offered a £15 voucher of their choice towards any costs of participation and in recognition of their time following the interview (£25 for attending the Feedback Workshop). Second (principle of valid consent^55^), it is likely that there will be low literacy and people for whom English is their second or even third language within the participating Traveller communities.^1^ ^2^ ^5^ The PIS for each community will be developed with our ‘Community Partners’ using simple language and images; and translated for Roma communities. Third (principle of valid consent^55^), according to Gillick competence,^56^ the teenage girls (under 16 years of age) could consent for themselves to take part in an interview about immunisation if they have sufficient understanding and intelligence to understand fully what is proposed. However, we are mindful that Traveller communities commonly remove their children from sex education classes in school,^57^ and so may be unwilling for their daughters to participate in a discussion about an immunisation to protect against infections transmitted through sexual behaviour (HPV vaccine). We will therefore seek assent from the girls themselves and consent from a parent.

### Outputs and dissemination

There will be three key outputs:

1. Comprehensive, in-depth findings on the barriers and facilitators to uptake and acceptability of childhood/adult immunisation both within and across six Traveller communities living in four cities across England and Scotland.
2. A prioritised list of potentially feasible and acceptable interventions for increasing immunisation uptake across the five levels of the SEM for these Traveller communities.
3. Methodological development in undertaking research with diverse Traveller communities living in different localities. Historically, research has been small scale, localised and with one community. Lessons learnt from this large-scale multi-city qualitative study can be used to improve the quality of future research in Traveller communities of a similar scale.

The findings will be published within the final report to the National Institute for Health Research Health Technology Assessment (NIHR HTA) programme. A local summary will be disseminated to the community and local organisations with whom we have worked to access/recruit Traveller participants to the study and to service providers in that locality who took part. A summary of the findings on the barriers and facilitators to uptake and acceptability of immunisation for all six Traveller communities will be sent to relevant national UK agencies and organisations responsible for the implementation of immunisation policy and Traveller health and welfare including professional bodies. We will also submit research papers to relevant professional journals and conferences. Academic papers will be submitted for publication in high-impact peer-reviewed journals.
